# Lake warming favours small-sized planktonic diatom species

**DOI:** 10.1098/rspb.2008.1200

**Published:** 2008-09-23

**Authors:** Monika Winder, John E. Reuter, S. Geoffrey Schladow

**Affiliations:** Tahoe Environmental Research Center, University of CaliforniaOne Shields Avenue, Davis CA 95616-8803, USA

**Keywords:** phytoplankton, climate change, long-term ecological research, *Cyclotella*, nutrients, stratification

## Abstract

Diatoms contribute to a substantial portion of primary production in the oceans and many lakes. Owing to their relatively heavy cell walls and high nutrient requirements, planktonic diatoms are expected to decrease with climate warming because of reduced nutrient redistribution and increasing sinking velocities. Using a historical dataset, this study shows that diatoms were able to maintain their biovolume with increasing stratification in Lake Tahoe over the last decades; however, the diatom community structure changed. Increased stratification and reduced nitrogen to phosphorus ratios selected for small-celled diatoms, particularly within the *Cyclotella* genus. An empirical model showed that a shift in phytoplankton species composition and cell size was consistent within different depth strata, indicating that altered nutrient concentrations were not responsible for the change. The increase in small-celled species was sufficient to decrease the average diatom size and thus sinking velocity, which strongly influences energy transfer through the food web and carbon cycling. Our results show that within the diverse group of diatoms, small-sized species with a high surface area to volume ratio were able to adapt to a decrease in mixing intensity, supporting the hypotheses that abiotic drivers affect the size structure of planktonic communities and that warmer climate favours small-sized diatom cells.

## 1. Introduction

Diatoms are abundant phytoplankton in aquatic habitats and are large contributors to carbon fixation in many lakes and the oceans (Smetacek 1999; Reynolds 2006). These unicellular algae are an essential link for energy transfer to upper trophic levels as they are a preferred high-quality food source for primary consumers (Brett & Muller-Navarra 1997). Diatoms are also important in aquatic biogeochemistry as they are the principal source of biologically induced carbon export from surface to deep waters and play a central role in nutrient cycling (Treguer 2002). Diatoms are characterized by silicon oxide cell walls, and individual cells are most commonly in the size range of 10–200 μm. As these relatively large cells with dense cell walls cause them to sink readily (Smol *et al*. 1984), many diatoms benefit from turbulent mixing to remain suspended in euphotic water layers (Huisman *et al*. 2004). Compared with other phytoplankton taxa, diatoms have relatively high growth rates and are generally adapted to low light levels in high nutrient waters (Reynolds 2006). These growth requirements can limit the temporal and spatial range of planktonic diatoms. In temperate systems, diatom population dynamics show strong seasonal patterns and often bloom in spring when growth conditions of mixing, nutrients and light availability are optimal (Reynolds 1984; Sommer 1989). Similarly, diatoms can form massive blooms in ocean areas with strong physical mixing (Chisholm 1992; Bopp *et al*. 2005), whereas their abundances are typically low in the oligotrophic stable open oceans (Boyd & Doney 2002). Experimental and modelling studies further substantiate that diatom abundances are linked to changes in the thermal–physical dynamics of the water column (Diehl 2002; Huisman *et al*. 2004). As thermal stratification and physical mixing processes are controlled by climatic forcing, it is expected that climate change will particularly affect diatom abundance and community structure (Smol *et al*. 2005; Rühland *et al*. 2008).

Climate and meteorological conditions alter phytoplankton dynamics through a chain of linked processes. The vertical thermal and, hence, density gradient affects the energy required to mix nutrient-depleted surface waters with the nutrient-rich deep waters during seasonal turnover. The presence of a density gradient also suppresses the formation of turbulence (Turner 1979), which increases sinking velocities of non-motile species (Smayda 1970). These physical changes of the water column affect the vertical distribution of algae (Fahnenstiel & Glime 1983) and therefore the growth environment of light and nutrients experienced by individual cells (Reynolds 2006). Climate warming increases the density gradient between the upper and lower water layers, which suppresses the upward flux of nutrients and reduces nutrient availability for autotrophic organisms in the euphotic zone (Verburg *et al*. 2003; Behrenfeld *et al*. 2006). Consequently, climate warming is expected to enhance the competitive advantage of cell types that are better competitors for nutrients (Falkowski & Oliver 2007) and that are able to maintain their vertical position in the euphotic zone (Huisman *et al*. 2004). As diatoms generally have high nutrient requirements and high sinking rates (Litchman *et al*. 2006; Reynolds 2006), it is expected that, with climate warming, diatoms will be replaced by other phytoplankton taxonomic groups best adapted to an environment with reduced turbulent mixing (Findlay *et al*. 2001; Boyd & Doney 2002; Bopp *et al*. 2005), and their seasonal dynamics is expected to be less predictable owing to intensified changes in nutrient fluxes and sinking rates (Huisman *et al*. 2006).

There is, however, considerable heterogeneity at the diatom taxon level as morphological and ecophysiological traits differ widely among species. Individual diatom cell size spans from 2 to 500 μm and cells have different shapes that affect form resistance and sinking rates (Smol *et al*. 1984; Round *et al*. 1990). Resource needs for individual species are not well characterized (Kilham *et al*. 1996); nevertheless, many major ecophysiological traits and sinking parameters of phytoplankton can be related to cell size and cell morphology (Litchman *et al*. 2007). For instance, nutrient uptake, growth and minimal nutrient quota are size dependent across phytoplankton taxa (Williams 1965; Litchman *et al*. 2006). These general characteristics are also consistent within the wide size range of diatoms: small-sized diatoms with high surface area to volume ratios have small diffusion boundary layers that enable efficient nutrient uptake; they have an inherently superior ability to harvest light, exhibit lower sinking rates and divide more rapidly compared with large-sized cells that have a low surface area to volume ratio (Litchman *et al*. 2006). By contrast, larger cells are more likely selected for when nutrients are resupplied by turbulent mixing (Chisholm 1992; Bell & Kalff 2001). As a result, diatom cell size is a powerful predictor of optimum dynamic performance (Reynolds *et al*. 2002) that summarizes major ecophysiological traits. These relationships are useful to scale up from nutrient physiology to the size structure of phytoplankton communities (Irwin *et al*. 2006), to explain modern phytoplankton spatial and temporal distribution patterns (Litchman *et al*. 2007), and have been used to reconstruct the evolutionary history of the phytoplankton nutrient environment (Finkel 2007). Thus, it can be expected that, as a taxonomic group, they may have the capacity to adapt to climate-induced mixing alterations by selecting for species that are best adapted to enhanced water column stratification.

In support of this hypothesis, fossil records document that the diatom community structure has been altered by environmental change over its evolutionary history (Finkel *et al*. 2005). On a geological time scale, macroevolutionary changes coincide with changing hydrodynamic mixing processes, such as change in the sea level and the ocean thermal structure that are climate driven (summarized in Falkowski & Oliver 2007). The marine diatom size structure and diversity shifted towards a smaller size in response to intensified thermal stratification linked to increasing ocean temperatures (Burckle *et al*. 1981; Finkel *et al*. 2005). Similarly, palaeolimnological studies from high-latitude and -altitude systems indicate a widespread expansion of pelagic and small-sized diatoms in more recent decades (Sorvari *et al*. 2002; Saros *et al*. 2003; Rühland & Smol 2005; Roberts *et al*. 2006), which is largely attributed to a longer ice-free season and increased stratification in deep lakes (Smol *et al*. 2005). Recently, Rühland *et al*. (2008) extended these palaeolimnological observations to include temperate lakes. Using a meta-analysis of over 200 palaeolimnological studies, they showed that increases in small-size *Cyclotella* species were a common occurrence in a large number of North American and European lakes, which could be linked to factors related to recent climatic warming over the last few decades. These observations from the past indicate that environmental factors, such as change in temperature and physical mixing, affect the competitive advantage of phytoplankton cells and can cause changes in the phytoplankton community structure.

In the present study, we use a long-term diatom dataset (1982–2006) from Lake Tahoe in conjunction with environmental variables to investigate the effects of temperature warming on the diatom community structure. Air temperatures in the Tahoe Basin experienced a warming trend over the last century (Cayan *et al*. 2001), which is linked to the subsequent increased Lake Tahoe water temperature and vertical temperature gradient (Coats *et al*. 2006; Winder & Hunter 2008). The present study shows that as a major taxonomic group, the diatoms in Lake Tahoe have collectively been able to maintain their biovolume in the presence of intensified stratification because species best adapted to changing mixing and nutrient regimes were able to flourish and expand.

## 2. Material and methods

### (a) Site description

Lake Tahoe is a subalpine lake located at an elevation of 1898 m.a.s.l. in the Sierra Nevada mountain range of California and Nevada, USA (39° N, 120° W). The lake has a surface area of 500 km^2^, a maximum depth of 501 m, a mean depth of 333 m and is free of ice the entire year. The lake starts to stratify in April and by October stratification begins to break down with a deepening of the thermocline; the average thermocline depth is 21 m in August and increases to 32 m in October (Coats *et al*. 2006). Lake Tahoe is oligotrophic and average annual total phosphorus concentrations in the top 100 m water were 7.4 μg l^−1^ (±1.7 s.d.) between 2001 and 2006. Available phosphorus (orthophosphate) concentrations were slightly elevated in the 1980s (ranging between 2.5 and 1.5 μg l^−1^) and were at a consistently low level of 1–1.5 μg l^−1^ thereafter (Winder & Hunter 2008). The annual average Secchi depth ranged between 24.7 and 19.5 m over the sampling period and is located above the euphotic zone depth, which ranged between 52 and 66 m (Winder & Hunter 2008), and the deep-water chlorophyll maximum typically develops during the summer between 40 and 60 m. The zooplankton community has been relatively consistent since the 1980s, dominated by the copepod species *Diaptomus tyrrelli* and *Epischura nevadensis*, and the rotifers *Kellicottia* spp., *Keratella* spp. and *Polyarthra* spp. Previous studies showed that top-down control of the copepod-dominated zooplankton community in Lake Tahoe is negligible (Elser & Goldman 1991). Land-use in Tahoe's drainage basin is highly regulated with reduced development and virtually no contributions from industry or agriculture; 87 per cent of the land area consists of undeveloped forest and other vegetative communities. Sewage was diverted from the basin in the late 1960s and current lake management strategies focus on controlling non-point sources of fine sediment (below 20 μm) and nutrients.

### (b) Data collection

Physico-chemical data used in this study were collected from a near-shore station (Index station, maximum depth of 125 m) and a mid-lake station (maximum depth of 460 m) (for a detailed map, see Jassby *et al*. 2003), with an average sampling interval of 12 and 26 days, respectively, between 1982 and 2006. Measurements of temperature at depths of 0, 2, 5, 10, 15, 20, 30, 50 and 100 m were combined from the near-shore and mid-lake stations. The data from the two sites have typically shown very little differences at the same depths (Coats *et al*. 2006). For each depth, daily values of temperature were interpolated using cubic spline interpolation. A daily temperature profile at 1 m intervals was then derived by linear interpolation, and used to calculate vertical density profiles. Density (*ρ*) was calculated as a function of temperature, salinity (assumed constant at 0.042 psu) and pressure (depth). The density difference between the mean density of 30–50 m and 0–10 m water was used as an indicator of stratification strength owing to detailed depth resolution of the historical temperature measurements in this depth layer compared with the lower water layers. The depth of spring mixing was determined from nitrogen profiles (described in Winder & Hunter 2008). Nitrate and phosphorus were measured at the near-shore station at 0, 2, 5, 10, 15, 20, 30, 40, 50, 60, 75, 90 and 105 m depths at a monthly interval over the period of record (nutrient concentrations at the mid-lake station are shown in Winder & Hunter 2008). Further details about nutrient measurements are given in Winder & Hunter (2008).

Samples for phytoplankton counting and identification were collected from 0, 2, 5, 10, 15, 20, 30, 40, 50, 60, 75, 90 and 105 m depths at the Index station between 1982 and 2006 and from 0, 10, 50 and 100 m at the mid-lake station between 1992 and 2006 and fixed with Lugol's solution (no phytoplankton data are available for 1990 and 1991). Average sampling frequencies for phytoplankton were 13 days at the Index station and 30 days at the mid-lake station. Species composition and abundances were similar for both stations (unpublished data), so the data from both stations were pooled for calculating monthly averages. Taxonomic phytoplankton identification and cell counts of species were analysed from a composite sample, which was composed of volume-weighted aliquots from each depth sample based on the hypsographic curve of the lake. In addition, discrete depth samples at 5, 20, 40, 60, 75 and 90 m were analysed once a month from 1983 to 1987 and 2002 to 2006. Phytoplankton cell identification, enumeration and biomass calculation are described in Winder & Hunter (2008). A mean cell volume was assigned for each phytoplankton species based on cell size measurements. In each sample, a minimum of 400 cells was counted and linear dimensions were measured for dominant individuals to account for any change in cell size. An average diatom cell volume as an indicator of size was estimated over the sampling period by correcting for the relative contribution of each species to total genus biovolume. Three size categories of the diatom community were distinguished based on diatom size-frequency distribution (see figure S1 in the electronic supplementary material) measured from maximum linear dimension (MLD): 4–15 μm (nano-diatoms); 15–40 μm; and above 40 μm (micro-diatoms). A list of diatom species identified in Lake Tahoe including cell size information is available in table S1 in the electronic supplementary material.

### (c) Modelling size-abundance spectra (SAS) of diatoms

An empirical model was used to relate the slope of the SAS to changes in the mixing regime. A linear regression model was applied to the octave (log_2_) scale of cell volume (*x*-axis, μm^3^) and the log-transformed values of relative cell abundance (*y*-axis) for each individual species (Rodriguez *et al*. 2001),

$$\text{log}[\text{cell}\;\text{abundance}\;(\text{cell}\;{\text{ml}}^{-1})]=a-b\;{\text{log}}_{2}[\text{cell}\;\text{volume}\;({\mu \text{m}}^{\text{3}})].$$

The slope (*b*) of the regression model was used as an indicator of diatom size structure. Phytoplankton counts from the discrete depth counts above (5 and 20 m) and below the thermocline (40, 60, 75 and 90 m) were pooled to calculate *b* for the respective depth layer and sampling date. Average slopes during the stratification period (May–October) were calculated including the regressions that were significant at *p*=0.05 (less than 2% of regression models were excluded owing to poor SAS fits).

### (d) Data analysis

Trends were estimated with robust non-parametric methods that are efficient in the presence of non-normal residuals and outliers. Monotonic trends in monthly series were assessed using the seasonal Kendall test (SKT) adjusted for serial correlation (Hensel & Hirsch 1992). To examine the effects of environmental changes on biomass of different diatom size categories, generalized linear models with a Gaussian error distribution were used. Model selection was based on a step-wise procedure and the improvement of the fit gained was assessed using the *Χ*^2^ change in deviance at the 5 per cent level. To identify the most parsimonious model, the Akaike information criterion (AIC) corrected for small sample size (AIC_c_) (Burnham & Anderson 2002) was used. Finally, the retained predictor variables were included in a model using generalized least squares (GLS) and the residuals were modelled as a first-order autoregressive term to account for serial correlation. Statistical analyses were performed using the statistical software R (R Development Core Team 2008) and S-Plus.

## 3. Results

### (a) Changes in the physical and chemical environment

**Figure 1:**
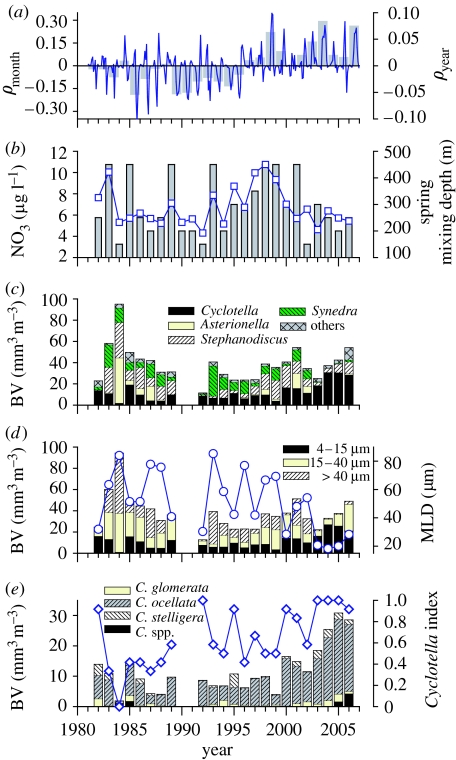
Long-term trends of physical, chemical and diatom trends from Lake Tahoe between 1982 and 2006. (*a*) Stratification strength is indicated by density difference anomalies (*ρ*, kg m^−3^) between lower and upper water strata. The solid line indicates the 12-month running average of deviations from the 1982–2006 monthly means; the grey bars indicate annual deviations. (*b*) Depth of spring mixing (grey bars) and annual average nitrogen concentration (open squares) in the upper 100 m water column. (*c*) Average annual diatom biovolume (based on composite samples between 0 and 105 m depth); the most dominant genera are highlighted. (*d*) Average annual diatom biovolume of different diatom size classes (bars) and average size (maximum linear dimension, MLD) of the diatom community (open circles). Size is corrected for the relative contribution of different taxa. (*e*) Annual biovolume average of the most dominant *Cyclotella* species (bars; for other *Cyclotella* species, see table S1 in the electronic supplementary material) and the *Cyclotella* index (open diamonds) reflects the relative time in each year when a bloom was detected (see text).

The warming trend in the Tahoe Basin over the last century affected the water temperature of Lake Tahoe and its mixing regime (Coats *et al*. 2006). Between 1982 and 2006, stratification increased (figure 1*a*), indicated by the increasing density difference between the lower and upper water layers (SKT: *τ*=0.49, *p*<0.001). The deep water of Lake Tahoe holds an excess supply of regenerated nitrogen (see figure S2 in the electronic supplementary material) and the depth of spring mixing is an important process for the redistribution of this nutrient to the euphotic zone. This is indicated by the significant correlation between spring mixing depth and annual mean nitrate concentration in the upper 100 m water column (*r*=0.62, *p*=0.001; figure 1*b*).

### (b) Shift in the diatom community structure

**Figure 2:**
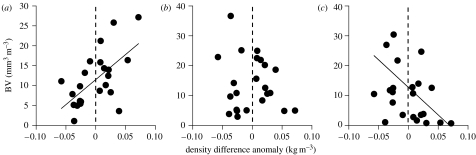
Relationship between annual diatom biovolume of different size fractions and stratification intensity anomalies (as indicated by the density difference anomaly) in Lake Tahoe between 1982 and 2006. A first-order autoregressive term is included to account for serial correlation. (*a*) 4–15 μm (*r*=0.61, *p*=0.02), (*b*) 15–40 μm (*r*=0.13, *p*=0.8) and (*c*) above 40 μm (*r*=−0.43, *p*=0.04).

Diatom community biovolume showed high interannual variability with average annual values ranging between 11.4 and 95.0 mm^3^ m^−3^ and no consistent trend over the sampling period according to seasonal trend statistics (*τ*=−0.07, *p*=0.36; figure 1*c*). There was, however, a shift in relative species contribution: *Synedra*, *Asterionella* and *Stephanodiscus* dominated the 1980s and 1990s and *Cyclotella* has been dominant since the year 2000. Overall diatom species in the size range between 4 and 15 μm increased significantly (SKT: *τ*=0.24, *p*=0.018), while intermediate-sized diatom biovolume was relatively consistent (SKT, *τ*=−0.11, *p*=0.3) and large-sized diatoms decreased (SKT: *τ*=−0.27, *p*=0.017) over the sampling period (figure 1*d*). The interannual change within the smallest size range (4–15 μm) was significantly related to stratification strength and biovolume was higher in years with higher stratification (figure 2*a*). The best-supported model for this size fraction further suggests an additive effect of nutrients and zooplankton density (table 1). The negative association with N : P ratios indicates that increased return of regenerated nitrogen from deep waters into the euphotic zone during intensive spring mixing decreases their competitive advantage over larger sized cells. Zooplankton density was negatively associated with this smaller size range, but was not significant after accounting for mixing strength and nutrient concentration. The year-to-year change in biovolume of the intermediate-sized diatom fraction was not correlated with stratification strength (figure 2*b*) and was best supported by additive effects of nitrogen and phosphorus concentrations (table 1). Both nitrogen and phosphorus increased annual biovolume of this size class. Stratification strength was the only significant predictor that supported interannual biovolume changes of the largest sized diatom fraction (above 40 μm; table 1), and biovolume within this size fraction was significantly higher in years with stronger turbulent mixing (figure 2*c*).

**Figure 3:**
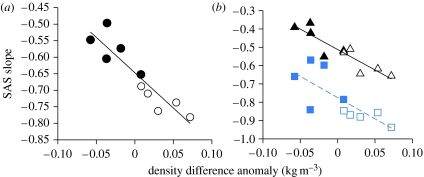
Slope of diatom size-abundance spectra (SAS slope) plotted against density difference anomalies (figure 1) as an indicator of stratification intensity for samples including (*a*) depth between 0 and 90 m (black filled circles, 1983–1987; black open circles, 2002–2006; GLS: *r*^2^=0.84, *p*<0.001) and (*b*) for the upper (0–30 m: blue filled squares, 1983–1987; blue open squares, 2002–2006; GLS: *r*^2^=0.56, *p*=0.02) and lower (40–90 m: black filled triangles, 1983–1987; black open triangles, 2002–2006; GLS: *r*^2^=0.82, *p*=0.001) water depth layers.

The dominance of small-sized cells in more recent years is also reflected in a shift towards a diatom community with a smaller cell size (SKT: *τ*=−0.31, *p*=0.01; figure 1*d*). Average annual diatom size decreased from approximately 67 μm in 1982 to approximately 35 μm in 2006, as indicated by a linear regression of diatom size versus year (*r*=−0.48, *p*=0.02). A detailed analysis of diatom species at specific depths indicated that the trend of increasing small-sized diatoms and decreasing larger sized cells was consistent within the nutrient-limited upper (0–30 m) and nutrient-rich lower (40–90 m) water strata (see table S2 in the electronic supplementary material). This is further supported by the slope of diatom SAS, which were more negative in recent years with intensified stratification compared with the 1980s (figure 3*a*). This trend was consistent in the upper and lower water layers (figure 3*b*; figure S3 in the electronic supplementary material), and thus with the vertical nutrient gradient. The effect, however, was more pronounced in the upper water layer, suggesting less selection pressure for small-sized cells in deep-water layers. In addition, diatom diversity did not change over the sampling period (SKT: *τ*=−0.12, *p*=0.26) and average diatom cell size did not correlate with diversity (*r*=0.11, *p*=0.63), indicating that the change in diatom cell size is not a response of changes in species diversity.

The increasing trend in small-sized diatoms was mainly accounted for by the increasing trend in *Cyclotella* spp., which was the only diatom genus that showed a significant increase over the sampling period (SKT *Cyclotella*: *τ*=0.32, *p*=0.002; for long-term trends of dominant genera, see figure S4 in the electronic supplementary material). These small-sized centric diatoms contributed over 50 per cent of the total diatom biomass in most recent years. Among the six *Cyclotella* species present in Lake Tahoe (see table S1 in the electronic supplementary material), the small-sized species *C. ocellata* (12 μm MLD) showed the strongest increase in biomass (SKT *τ*=0.31, *p*<0.001), followed by *C. glomerata* (4.4 μm MLD; *τ*=0.22, *p*=0.01; figure 1*e*). Numerically, the smallest sized *Cyclotella* species present in Lake Tahoe (*C. glomerata* and *C. stelligera*, 6.6 μm MLD) dominated and were up to more than 70 per cent of total cell numbers in recent years. The fraction of time in a year when *Cyclotella* blooms were detected (a bloom is defined when *Cyclotella* contributes more than 45 per cent to total diatom abundance) increased over the period of record and reached unity in recent years, indicating that *Cyclotella* dominated the diatom community throughout the year (figure 1*e*).

## 4. Discussion

Shifts in size structure have been observed in planktonic organisms, including diatoms, dinoflagellates and foraminifera over geological and centennial time scales (Schmidt *et al*. 2004; Smol *et al*. 2005; Finkel *et al*. 2007; Rühland *et al*. 2008), and these shifts have been linked to altering water column stratification related to changing climate. The present study shows that contemporary climate warming is exhibiting a selection pressure on diatom cell size in the same direction as was observed in palaeoecological studies. In both cases, this favours small-sized diatoms that are able to outcompete larger sized cells and expand under intensified stratification. Our observation corroborates palaeoecological research findings (Arrhenius 1952; Smol *et al*. 2005; Falkowski & Oliver 2007), suggesting that climate interacts with diatom communities through effecting mixing and thereby changing cell sinking velocities and the rate at which nutrients are redistributed in the water column.

Lake Tahoe's temperature and stratification trend over the last four decades are similar to trends in other freshwater systems (Coats *et al*. 2006; Keller 2007) and the oceans (Bopp *et al*. 2005) in response to recent climate warming and are widely predicted under future climate scenarios. The diatom community in Lake Tahoe responded to changes in the physical environment by favouring specific cell types, which is in accordance with size-based traits associated with this major taxonomic group (Litchman *et al*. 2007). Under more intensified stratification in more recent years, small-sized diatom cells increased, whereas large-sized diatoms dominated under stronger turbulent mixing conditions and decreased over the record of sampling. Moreover, the consistent trend of a more negative slope of the SAS within both the upper and lower water layers in recent years gives support to the existence of a direct vertical motion control (Rodriguez *et al*. 2001). If the diatom size structure in Lake Tahoe was affected primarily by change in nutrients due to external inputs, we would expect that the diatom size structure would stay consistent in the deep strata near the nitrocline with sufficient nutrient supply. However, phosphorus concentrations changed only slightly in Lake Tahoe over the sampling period and smaller sized cells increased also in the lower water layers with higher nutrient supply. This suggests that the shift towards smaller sized cells is a function of changing physical conditions and supports other observations that abiotic fluctuations play an important role in maintaining algal structure in natural communities (Descamps-Julien & Gonzalez 2005).

Vertical mixing largely controls interannual nutrient fluxes to the upper water layer in deep lakes and this flux path is particularly important for Lake Tahoe, where both nitrogen and phosphorus are limiting phytoplankton growth (Goldman *et al*. 1993; Reuter *et al*. 2003). Consequently, reduced mixing probably resulted in a more nutrient-depleted chemical environment that favoured small-sized diatom cells in Lake Tahoe. The morphometric shift in diatoms under these conditions is largely due to the presence and expansion of small-sized, fast-growing centric diatoms of the genus *Cyclotella* that have high surface area to volume ratios. This genus is known to be a good competitor for nitrogen and is often favoured over other diatom species under conditions of strong summer stratification (Bradbury 1988; Tolotti *et al*. 2007). Increases in *Cyclotella* were particularly pronounced in the years between 2002 and 2006 when N : P ratios were low (Winder & Hunter 2008) and stratification high. Palaeolimnological studies showed a widespread increase in *Cyclotella* species over the last several hundred years in a number of freshwater systems (Saros *et al*. 2003; Harris *et al*. 2006), a trend largely attributed to climate warming and an associated decreased ice cover and increase in water column stability (Rühland *et al*. 2003, 2008; Rühland & Smol 2005). Palaeorecords provide important information for recognizing long-term trends but the causal linkages are inferential, and precise information on the causes of the change is often not observable from these data. The present study confirms that *Cyclotella* are good competitors under stable and nutrient-diluted conditions, and their success is largely linked to climate-induced changes in the physical dynamics of the water column. Particularly, small-sized species within this genus were able to expand over the entire season under nutrient-depleted environments, caused by intensified stratification.

The absolute number of small-sized *Cyclotella* species in Lake Tahoe may be a reflection of a shorter life cycle and increased growth rates (Jewson 1992). Diatom growth rates vary largely among species (Reynolds 2006); however, in general, small-sized cells, such as *C. glomerata* and *C. stelligera*, have higher intrinsic growth rates than larger sized cells (Litchman *et al*. 2007). By contrast, larger sized species such as *Stephanodiscus* spp. (approx. 21 μm) and *Asterionella* sp. (approx. 87 μm) found in Lake Tahoe were favoured in the 1980s and late 1990s, when intensive spring mixing resulted in higher nitrogen supply. Cell size and surface area to volume relations also influence sinking speed and, according to the Smayda model (Smayda 1970), the sinking speed of the diatom community between 2002 and 2006 was approximately 0.85 m day^−1^ (mean size 18 μm, assuming that cells are spherical and do not coagulate) compared with approximately 3.3 m day^−1^ (mean size 78 μm) between 1983 and 1985. In addition to cell size and shape, sinking rates are also influenced by cell physiology and light conditions (Tilman & Kilham 1976), such as sinking rates decrease under more favourable environments (Saros *et al*. 2005).

There was no evidence for a species-specific phenotypic response to intensified stratification in Lake Tahoe as cell size of individual species remained fairly constant over the sampling period (average coefficient of variation was 0.1±0.2 as indicated for 130 individual species). Through the asexual life cycle, the size of daughter cells decreases as each valve of a frustule produces a smaller, complementary valve resulting in a substantial decrease in cell size over several generations (Round *et al*. 1990). The original cell size is restored by sexual reproduction, which is cued to a minimum size range of a percentage decrease of the initial cell size (Edlund & Stoermer 1997). It is likely that there are species-specific differences (Burckle & McLaughlin 1977) and that size of individual species may have declined over time, although a decrease in cell size due to a phenotypic response would be minimal compared with changes in size among species. The upper size threshold for sexual reproduction in diatoms provides no obvious causal relationship for a phenotypic response and suggests that the increase in small-celled diatoms under stronger vertical stratification is associated with species replacement and a shift towards taxonomic small-sized diatom species in Lake Tahoe.

An alternative explanation for the increasing trend towards small-sized diatoms with intensified vertical stratification could be a response to decreasing water clarity. Huisman & Sommeijer (2002) demonstrated that in addition to water column mixing, the depth of light penetration influences phytoplankton size composition because phytoplankton cells need to remain in the euphotic zone for a sufficiently long period of time to proliferate. By means of theory and empirical data, they showed that cells with high sinking velocities can be sustained in transparent waters with a larger euphotic zone, whereas, in turbid waters, cells with low sinking velocities are at a competitive advantage. In Lake Tahoe, even though water clarity measured by the Secchi depth has decreased by nearly 10 m since 1968 (Goldman 1988; Jassby *et al*. 2003), observations showed that the rate of clarity decline has actually lessened since 2000 (TERC 2008), i.e. during the time period when the 4–15 μm sized species of *Cyclotella* became dominant. Since the euphotic zone depth (approx. 60 m) expands well below the Secchi depth, the depth of maximum primary productivity (between 10 and 40 m) and the summer thermocline depth (between 21 and 32 m) in Lake Tahoe, there is no strong reason to believe that changes in water clarity affected phytoplankton community structure to the extent reported in the study by Huisman & Sommeijer (2002). It is likely that the change in water clarity had some effect on the increase of small-sized diatoms inasmuch as small *Cyclotella* cells had a competitive advantage over larger sized cells with decreasing clarity; however, since Secchi depth was not a significant predictor of change in diatom size classes (table 1), our results suggest that intensified vertical stratification was the main driver of the change in diatom size structure in Lake Tahoe.

A possible floristic shift to fast-growing centric diatoms can have cascading ecosystem effects. Phytoplankton cell size of the pelagic ecosystem strongly influences the ecosystem's response to an increase in water column stratification. Phytoplankton cell size defines metabolic activity, growth rates, numerical abundance (Chisholm 1992; Reynolds 2006; Litchman *et al*. 2007) and sinking velocities (Smayda 1970), and is positively correlated with genome size (Oliver *et al*. 2007). Consequently, climate-driven changes probably alter important ecosystem functions, including biogeochemical cycling (Richardson & Jackson 2007), plankton community structure via size-dependent species interactions (Sommer *et al*. 2002) and evolutionary processes. Diatom minimum size is constrained to a spherical diameter of approximately 2–4 μm, owing to the unique properties of the silica frustule and cellular requirements (Raven 1994). Because of these restrictions, diatoms may be less competitive under stable and nutrient-depleted conditions such as in the stable open-ocean environments. Future intensifying stratification and reduction of deep spring mixing events may result in a replacement of diatom species with other phytoplankton cell types best adapted to reduced water column mixing.

## Figures and Tables

**Table 1:** Summary statistics of the most parsimonious model predicting annual biovolume of three diatom size categories in Lake Tahoe between 1982 and 2006 (no data were collected in 1991 and 1992) using GLS. The models were selected using AICc. (The full model is as follows: stratification intensity (stratification)+spring mixing depth+thermocline depth+Secchi depth+nitrogen+phosphorus+N : P molar ratio (N : P ratio)+zooplankton density (zooplankton).)

size category predictor	4–15 μm			15–40 μm			>40 μm		
		
	value	*t*	*P*	value	*t*	*P*	value	*t*	*P*
intercept	20.45±0.08	172.8	<0.001	−9.3±9.86	0.93	0.36	12.2±2.58	4.72	<0.001
stratification	165.01±0.08	21.08	<0.001		—	—	−168±79	2.14	0.04
nitrogen	—	—	—	−2.75±1.06	2.6	0.2	—	—	—
phosphorus	—	—	—	−3.44±3.26	1.05	0.3	—	—	—
N : P ratio	−0.30±0.08	3.77	0.001	—	—	—	—	—	—
zooplankton	−0.01±0.005	1.53	0.14	—	—	—	—	—	—
AICc	141.48			153.84			171.57		

## Supplementary Material

Additional figures and tables Table 1. Diatom species list and size dimensions. Diatom species list and size dimensions (maximum linear dimension, MLD) identified in Lake Tahoe between 1982 and 2006. Table 2. Trends of diatom biovolume size categories. Trend statistics of monthly diatom biovolume of three size categories within the upper (0−30 m) and lower (40−90 m) depth strata in Lake Tahoe between 1982 and 2006. *n*=12. Trends are expressed as the Seasonal Kendall's Tau test. Changes in the small and large-size fraction showed a significant trend at *p*<0.07 (*p*-values are corrected for serial correlation). Figure 1. Histogram of diatom species counts. Histogram of diatom species counts based on size (maximum linear dimension) in Lake Tahoe between 1982 and 2006. Figure 2. Vertical profiles in Lake Tahoe. Typical vertical profiles (0−400 m) of nitrogen, phosphorus (THP), temperature, and chlorophyll a during (a) the summer stratification (April–October) and (b) the winter mixing (November–March) season in Lake Tahoe in 2005/06. Chlorophyll a is measured in situ to ˜140 m depth. The deep water of Lake Tahoe holds an excess supply of regenerated nitrogen. Figure 3. Slopes of diatom Size-Abundance-Spectra in different depth. Box-plots of the slopes of diatom Size-Abundance-Spectra in different depth strata from 1983–1986 and 2002–2006. Slopes between the two periods were statistically different in the upper (ANOVA 0−30 m: F(1,8)=8.96, *p*=0.017) and deep (ANOVA 40−90 m : F(1,8)=11.54, *p*=0.009) water layer and over the entire water column (ANOVA 0−90 m : F(1,8)=35.53, *p*<0.001)
