# Supplementary material for: Lake warming favours small-sized planktonic diatom species
Source: Proc Biol Sci. 2008 Sep 23;276(1656):427–35. doi: 10.1098/rspb.2008.1200 (PMC2581674; doi:10.1098/rspb.2008.1200)
Supplement: Supplementary file 1 [file rspb20081200s03.doc]

**Electronic Supplementary Material**

**Supplementary Table 1.** Diatom species list and size dimensions (maximum linear dimension, MLD) identified in Lake Tahoe between 1982 and 2006.

**Supplementary Table 2**. Trend statistics of monthly diatom biovolume of three size categories within the upper (0 – 30 m) and lower (40 – 90 m) depth strata in Lake Tahoe between 1982 and 2006. N = 12. Trends are expressed as the Seasonal Kendall’s *Tau* test. Changes in the small and large-size fraction showed a significant trend at *P* < 0.07 (*p*-values are corrected for serial correlation).

|  |  | Trend; *P*-value |  |
| --- | --- | --- | --- |
| Depth strata | 4 - 15 µm | 15 - 40 µm | > 40 µm |
| 0 - 30 m | 0.30; *P* = 0.05 | -0.06; *P* = 0.61 | -0.34; *P* = 0.03 |
| 40 - 90 m | 0.32; *P* = 0.03 | -0.08; *P* = 0.57 | -0.35; *P* = 0.07 |

**Supplementary Figure 1.** Histogram of diatom species counts based on size (maximum linear dimension) in Lake Tahoe between 1982 and 2006.

**Supplementary Figure 2.** Typical vertical profiles (0 - 400 m) of nitrogen, phosphorus (THP), temperature, and chlorophyll *a* during (a) the summer stratification (April – October) and (b) the winter mixing (Nov – Mar) season in Lake Tahoe in 2005/06. Chlorophyll *a* is measured *in situ* to ~140 m depth. The deep water of Lake Tahoe holds an excess supply of regenerated nitrogen.

**Supplementary Figure 3.** Box-plots of the slopes of diatom Size-Abundance-Spectra in different depth strata from 1983 – 1986 and 2002 – 2006. Slopes between the two periods were statistically different in the upper (ANOVA 0 – 30 m : *F*_(1,8)_ = 8.96, *p* = 0.017) and deep (ANOVA 40 – 90 m : *F*_(1,8)_ = 11.54, *p* = 0.009) water layer and over the entire water column (ANOVA 0 - 90m : *F*_(1,8)_ = 35.53, *p* < 0.001).

**Supplementary Figure 4.** Long-term trends of diatom genera biovolume between 1982 and 2006 in Lake Tahoe. Genera that appeared only sporadic over the sampling period are not included. Trends are expressed according to the Seasonal Kendall test adjusted for serial correlation.
